# Permeation Challenges of Drugs for Treatment of Neurological Tuberculosis and HIV and the Application of Magneto-Electric Nanoparticle Drug Delivery Systems

**DOI:** 10.3390/pharmaceutics13091479

**Published:** 2021-09-15

**Authors:** Sinaye Mhambi, David Fisher, Moise B. Tchoula Tchokonte, Admire Dube

**Affiliations:** 1 Discipline of Pharmaceutics, School of Pharmacy, University of the Western Cape, Cape Town 7535, South Africa; 4057837@myuwc.ac.za; 2 Department of Medical Bioscience, University of the Western Cape, Cape Town 7535, South Africa; dfisher@uwc.ac.za; 3 Department of Physics & Astronomy, University of the Western Cape, Cape Town 7535, South Africa; mtchokonte@uwc.ac.za

**Keywords:** magneto-electric nanoparticles, electroporation and drug delivery, blood–brain barrier and infectious disease, CNS, TB and HIV

## Abstract

The anatomical structure of the brain at the blood–brain barrier (BBB) creates a limitation for the movement of drugs into the central nervous system (CNS). Drug delivery facilitated by magneto-electric nanoparticles (MENs) is a relatively new non-invasive approach for the delivery of drugs into the CNS. These nanoparticles (NPs) can create localized transient changes in the permeability of the cells of the BBB by inducing electroporation. MENs can be applied to deliver antiretrovirals and antibiotics towards the treatment of human immunodeficiency virus (HIV) and tuberculosis (TB) infections in the CNS. This review focuses on the drug permeation challenges and reviews the application of MENs for drug delivery for these diseases. We conclude that MENs are promising systems for effective CNS drug delivery and treatment for these diseases, however, further pre-clinical and clinical studies are required to achieve translation of this approach to the clinic.

## 1. Introduction

TB is an infectious disease caused by the bacillus *Mycobacterium tuberculosis* *(M. tb).* Typically, TB affects the lungs but can spread and affect other sites of the body, i.e., extrapulmonary TB [1]. TB in the CNS is referred to as CNS TB and is one of the less common yet highly devastating mycobacterial infections in humans [2,3]. CNS TB is differentiated from TB meningitis (TBM) in that CNS TB begins as small tuberculous foci (Rich foci) in the brain, spinal cord or meninges [2], whereas TBM is a form of meningitis characterized by *M. tb* induced inflammation of the meninges. The location of the foci and the sites in which they are found determines their ability to be controlled as well as the type of CNS TB that occurs [2]. In 2019, the global burden of new cases of TB was estimated to be 10.0 million of which 8.2% resulted from concomitant HIV-TB co-infections [4]. In that same year, an estimated 1.2 million HIV-negative people died from TB and an additional 208,000 deaths occurred among HIV positive people [4]. Two-thirds of the global TB incidence in 2019 was accounted for by eight countries led by India (26%), followed by Indonesia (8.5%), China (8.4%), the Philippines (6.0%), Pakistan (5.7%), Nigeria (4.4%), Bangladesh (3.6%) and South Africa (3.6%) [4]. CNS TB accounts for 1% to 2% of active TB cases of which 15% to 40% of patients diagnosed with CNS TB die or become disabled even after anti-TB drug therapy [5]. Children and adolescents are more prone to meningitis involvement as the clinical presentation, compared to adults (patients older than 15 years of age) [6]. This highlights the dire state of the prognosis of CNS TB. A major challenge in the treatment of CNS TB is the delivery of drugs across the BBB, where the endothelial cells of cerebral capillaries provide a highly regulated interface between the peripheral circulation and the CNS. Paracellular tight junctions between the endothelial cells prevent ions, molecules and unwanted cells from passively entering the brain [7]. Most of the anti-TB drugs are unable to penetrate the BBB in sufficient amounts to effectively eradicate TB from the CNS. For example, isoniazid and pyrazinamide exhibit good cerebrospinal fluid (CSF) penetration, while the concentrations of rifampicin in the CSF may not reach the minimum inhibitory concentration for TB [8,9]. Ethambutol penetrates the CSF poorly [8]. The case is similar to CNS HIV.

HIV-1 entry into the CNS is largely mediated through blood lymphocytes and monocytes that enter perivascular spaces either during their natural surveillance or due to attraction to sites of inflammation by chemokines [10,11]. Alternatively, HIV can enter the CNS through lymphocytes which retain viruses that replicate in macrophages or as free virions where the mode of entry is through endothelial cells [10,12]. In the brain, HIV-1 resides in the perivascular macrophages, microglial cells and astrocytes [13,14,15]. Microglial cells are the main reservoirs of HIV-1 in the CNS [14]. The buildup of HIV in the CNS can lead to viral recurrence and rebound infection [16]. CNS HIV is associated with acute neurological symptoms similar to viral meningoencephalitis (the inflammation of the brain and its surrounding protective membranes) and leads to a high CSF viral load, local immune activation, changes in magnetic resonance imaging (MRI), and partially reversible neurocognitive impairment in some patients [17,18,19]. The inflammatory mediated disturbances increase months following the onset of neuroinvasion and lead to chronic neuronal damage [20]. Early initiation of antiretroviral therapy (ART) can hinder the formation of HIV-1 in the cells of the CNS and may further reduce immune activation and inflammation that are responsible for the spread of CNS infection [21]. HIV-positive individuals with TB are five times more likely to have CNS TB than HIV-negative individuals [22]. More than 40% of patients diagnosed with HIV develop complications of the CNS [23]. Current ARTs in clinical practice are also accompanied by delivery challenges across the BBB [16], resulting in an inefficacious elimination of HIV from the brain and the formation of HIV reservoirs in the brain. Tenofovir, nelfinavir, ritonavir, atazanavir, didanosine, stavudine and lamivudine are among the ARTs with the poorest CNS penetration [24,25]. Zidovudine, abacavir, lopinavir/ritonavir, atazanavir, nevirapine, lopinavir and indinavir have good CNS penetration and are among the favorable ARTs for treatment of CNS HIV [24,26], however, the use of these ART drugs has declined significantly as they are associated with high toxicity.

MENs are a class of NPs that exhibit magnetic and electric properties that can be controlled using magnetic and electric fields [27,28,29]. These NPs can enhance transient permeability of the BBB through nanoelectroporation of the cells. Nanoelectroporation is the use of focused electric pulse to porate the cell membrane to form a nanopore and also provides electrophoretic mobility of charged drug/gene molecules and/or NPs to move into the cell. Similar to other magnetic NPs, MENs have a nonzero saturation magnetization that could enable them to be guided throughout the body by the application of magnetic field gradients [29,30]. Subsequently, these NPs could be localized using traditional image-guided magnetic processes such as MRI and magnetic particle imaging [29] to facilitate drug delivery across the CNS.

This review discusses CNS TB and HIV drug delivery challenges, approaches to facilitating drug transportation into the CNS and MENs as delivery systems to improve drug penetration into the CNS for TB and HIV. Recommendations for future studies to address current knowledge gaps in the therapeutic application of MENs are made to advance the translation of this approach to the clinic.

## 2. Drug Permeation Challenges in CNS TB Treatment

The World Health Organization (WHO) guidelines suggest treatment of TBM with two months of rifampicin, isoniazid, pyrazinamide and ethambutol followed by ten months of rifampicin and isoniazid for all patients [31]. This guideline is based on the treatment guideline to treat pulmonary TB, which has been noted to not take into account the limited ability of anti-TB drugs to penetrate the BBB [9]. Only lipophilic drugs can readily penetrate the BBB via lipid-mediated free diffusion provided that the drug has a molecular weight (*Mw*) <400 g/mol and forms less than 8 hydrogen bonds [32]. Some antimicrobials (small hydrophilic molecules) like isoniazid and pyrazinamide are water-soluble agents and can cross the BBB paracellularly instead of transcellularly as seen with lipophilic agents [33]. Optimal BBB penetration is achieved when the logP values of drugs are in the range of 1.5 to 2.7, with a mean value of 2.1 [34]. Isoniazid is hydrophilic (*Mw* 137.14 g/mol) and is therefore able to penetrate the BBB freely [35,36] and 80% to 90% of isoniazid penetrates the CSF [9]. Isoniazid has proven potent bactericidal activity [9,37]. The CSF penetration of anti-infectives in humans predicted by the ratio of the area under the concentration-time curve in CSF to that in serum (*AUC_CSF_*/*AUC_S_*) [38]. Isoniazid has a logP value of −0.70 and is among some of the anti-infectives that achieve an AUC_CSF_/AUC_S_ ratio close to 1.0 and is, therefore, a valuable drug for the treatment of CNS infections with susceptible pathogens [39]. Rifampicin does not penetrate the BBB well. Rifampicin has a *Mw* of 822.9 g/mol and thus exceeds the *Mw* of 400 g/mol recommended to penetrate the BBB [40]. Rifampicin is however highly lipophilic (logP value of 2.7), and 80% of rifampicin is bound to plasma proteins [41]. Concentrations of rifampicin in the CSF are only 10% to 20% of those present in the plasma [9,42]. Thus, in plasma, most of the rifampicin is protein-bound and only the unbound portion is active, while in the CSF, very little protein is bound [9]. Pyrazinamide is a hydrophilic therapeutic agent with a *Mw* of 123.11 g/mol and a logP value of −0.6 [43]. Pyrazinamide has good CSF penetration (90–100%) [9]; concentrations of pyrazinamide in the CSF are close to that of serum [9,42]. Approximately 10% of pyrazinamide is protein-bound [43].

The inclusion of ethambutol in the CNS TB treatment regimen has been debated as this drug exhibits the poorest BBB penetration even when the BBB is inflamed [9]. Ethambutol (*Mw* 204.31 g/mol and logP value of −0.14), has CSF penetration of 20−30% [9,44]. Increasing the dose and duration of treatment with ethambutol is not advised as this may lead to visual and neurological disturbances. Increased serum uric acid levels and acute gouty arthritis have been reported from the increased use of ethambutol [45].

There is, however, not enough information on how these drugs cross the BBB and the exact concentration-time profiles of these drugs in the CSF are also not clear.

### 2.1. Drug Permeation Challenges in CNS HIV

The recommended treatment for CNS HIV is ART targeted to treat identified CNS HIV resistance mutations or empiric therapy using high CNS penetration drugs [46]. Intensified treatment with chemokine receptor 5 (CCR5) inhibitors is considered as add on therapy to reduce neuroinflammation if CNS HIV has not been detected [46]. The entry of antiretrovirals into the CNS is also influenced by their physicochemical properties [25]. High *Mw* antiretrovirals penetrate the CNS poorly [25]. Nucleoside analogues such as zidovudine, lamivudine, didanosine, stavudine and abacavir have *Mw* < 500 Da, low protein binding and can therefore penetrate the CNS well [25,47]. Abacavir (*Mw*: 286.33 g/mol) and zidovudine (*Mw*: 267.24 g/mol) demonstrate good CNS penetration than enfuvirtide (*Mw* exceeding 4000 Da), a fusion inhibitor, that penetrates the CNS poorly [25]. Nearly 35% of concentrations of abacavir in plasma penetrate the CSF [47]. Zidovudine, lopinavir/ritonavir and indinavir have logP values of 0.05, 1.7, 1.2 and 0.9 [48,49], respectively, indicative of good CNS penetration and are among the favorable ARTs for treatment of CNS HIV [26], however, the use of these ART drugs has declined significantly as they are associated with high toxicity. As discussed for TB drugs, having a low *Mw* and low protein binding does not ensure high CNS penetration. Tenofovir has low (5%) CNS penetration despite having a low *Mw* (287.213 g/mol) and low protein binding [47]. CSF concentrations of tenofovir are less than the in vitro inhibitory concentration to suppress 50% viral replication (*IC_50_*) for most patients and are assumed to be transported in the CSF via active transport [47]. Protease inhibitors have a *Mw* of 500 Da and demonstrate more than 90% protein binding, except for indinavir, which is less than 60% protein-bound in plasma [47]. The low protein binding efficiency of indinavir explains the higher total drug concentrations in the CSF compared to other protease inhibitors [47]. Nevirapine has a low *Mw* of 266.6 g/mol and is the least protein-bound non-nucleoside reverse transcriptase inhibitor (NNRTI) (60% protein binding) [47,50]. Nevirapine has a logP value of 2.9 that further indicates that it is a very lipophilic drug and can cross the BBB and arrive at the CNS [51]. In addition, nevirapine has good oral bioavailability (93 ± 9%) and a long half-life (45 h) [50]. On the contrary, the use of less toxic ART drugs with poor CNS penetration (tenofovir and atazanavir/ritonavir) has increased [26]. Although ARTs can improve symptoms of dementia and reduce viral load, the secondary effects of some ARTs such as abacavir, efavirenz, stavudine and zidovudine are associated with neurological disorders which further affect patient adherence to therapy [52]. Antidepressants, antipsychotics, and stimulants can be used to treat symptoms of depression, psychosis and lethargy, respectively. However, most treatment guidelines do not have special considerations regarding CNS HIV. Considering that the brain is one of the reservoirs of the virus, clearance of HIV from these reservoirs could lead to a cure for CNS HIV [13,15].

### 2.2. Drug Delivery across the BBB

Delivery of drugs to the CNS via the systemic route falls under two categories, i.e., invasive where deliberate access to the body is gained via incision, which may cause several complications such as damage to neurons and inflammatory reactions [53]. Invasive approaches require implantation of the devices such as osmotic pumps and depot formulations which therefore requires surgery and a sterile environment [53]. These techniques are therefore not suitable for chronic disease therapy such as in the case of HIV and TB.

**Figure 1:**
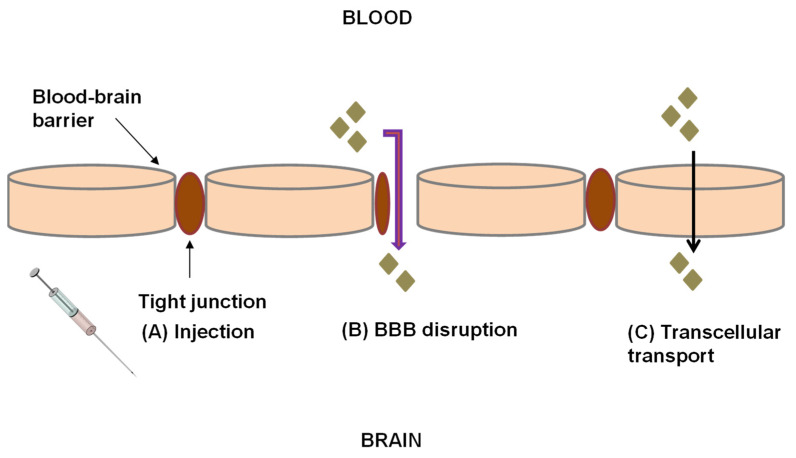
Strategies for crossing the BBB to deliver drugs. (**A**) Invasive approach: injection into the parenchyma of the brain is used to avoid the BBB when treating patients. (**B**) BBB disruption is achieved by injection of chemical agents, osmolytes or via focused ultrasound. This technique causes loosening of the tight junctions of the endothelium, permitting access to the brain by conventional drugs. (**C**) Transcellular transport of drug molecules through a cell.

Injection of drugs into the parenchyma of the brain (Figure 1A) is another example of an invasive technique. Disrupting the BBB with a hypertonic solution such as mannitol or using compounds such as bradykinin involved in the regulation of brain endothelial cellular junction can facilitate drug delivery to the brain [54,55]. Temporal disruption of the BBB can be achieved through the infusion of hyperosmolar solutions of arabinose, saline, mannitol or urea into the internal carotid artery. This leads to a shrinkage of endothelial cells resulting in the formation of gaps in the endothelial junction [53]. Le and Blakley [56] delivered gentamicin into the CSF of in-vivo guinea pigs by causing temporal disruption of the BBB through the administration of mannitol. The results showed that the rate of entry and exit of gentamicin was increased by mannitol through the blood labyrinth barrier significantly (*p* = 0.0044). The pharmacokinetic models for gentamicin showed no significant differences between the model without gentamicin and the model with gentamicin and mannitol (*p* = 0.433). This finding indicated that renal clearance of gentamicin from the blood was not altered by mannitol. The concentration of gentamicin in CSF and perilymph was always remarkably lower than that in blood [56]. Focused ultrasound is another invasive delivery method of agents across the BBB. Focusing ultrasound in the area of interest with low intensity allows the reversible disruption of the BBB at target sites. There are currently no studies on the delivery of drugs for TB or HIV in the brain using focused ultrasound. Yang et al. [57] developed an in vitro intracranial brain tumor model in NOD-*scid* mice using human brain glioblastoma multiforme 8401 cells to effectively deliver human atherosclerotic plaque-specific peptide-1 (AP-1)-conjugated liposomes containing doxorubicin (AP-1 Lipo-Dox) across the BBB. Yang and coworkers [57] utilized pulsed high-intensity focused ultrasound (HIFU) to disrupt the BBB transcranially by delivering ultrasound waves in the presence of microbubbles. The authors reported that animals receiving drugs followed by pulsed HIFU presented with a significant accumulation of the drug in the tumor cells compared to control animals treated with injections of AP-1 Lipo-Dox or unconjugated Lipo-Dox [57]. Focused ultrasound may be problematic in that it may cause subtle and elusive damage to DNA [58], may be time-consuming to administer, and may induce apoptosis [59]. However, BBB disruption (Figure 1B) is not a logical method of drug delivery. This technique causes loosening of the tight junctions of the endothelium, allowing the transport of unwanted toxins into the brain in addition to the drugs.

Noninvasive approaches do not involve incision of the BBB, nor do they cause permanent alterations in the integrity of the BBB. Examples of non-invasive approaches that have been used for the delivery of drugs across the BBB include altering the solubility of the drug, NP drug delivery systems, chimeric peptides, enhanced transcellular transport, transport/carrier systems, Trojan horse approach, intranasal delivery, monoclonal antibody fusion proteins, peptidomimetics, immunophilins, efflux transporter inhibitors and prodrug approaches [53,60] (Table 1).

**Figure 2:**
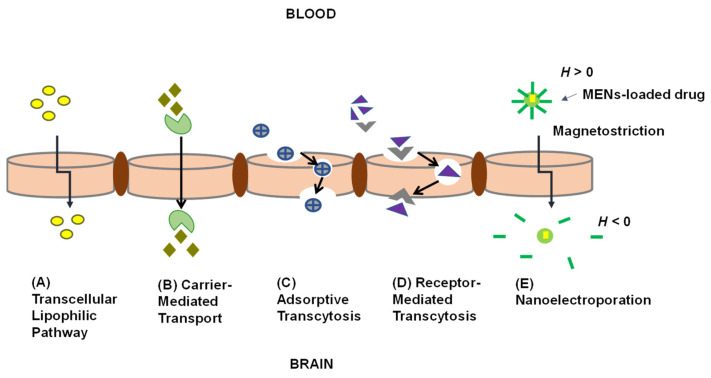
Illustration of transport pathways for BBB transport. (**A**) The transcellular lipophilic pathway allows the passive diffusion of small, lipophilic molecules across the BBB and into the brain. (**B**) Carrier-mediated transport employs specific proteins to move molecules from the environment into and through the cell. (**C**) Endocytosis of molecules via adsorptive transcytosis nonspecifically and transported through the cell. (**D**) Specific ligands bind receptors and are endocytosed and transported through the cell via receptor-mediated transcytosis. (**E**) Translocation of MENs across the BBB. Once MENs have been administered into the body, an externally applied magnetic field changes the shape of the inner core. Magnetostriction, changes the shape of the piezoelectric shell. The magnetic field induces the nanoelectroporation of the diseased cell (see Section 2.3).

Chimeric peptides are a category of peptides and protein molecules that are coupled with suitable vectors [53]. The ability of chimeric peptides to impart properties from each “parent” protein to the subsequent chimeric protein has enabled their use in drug delivery [53]. Chimeric peptides are formed by the covalent coupling of a non-transportable peptide (e.g., beta-endorphin) to a transportable peptide that undergoes receptor- or absorptive-mediated transcytosis at the BBB [61]. Immunophilins are involved in processes such as protein folding, protein trafficking, receptor signaling, and transcription [62] and these compounds display biological functions when complexed with their ligands [62]. Immunophilins are comprised of a family of conserved proteins which contain binding abilities to immunosuppressive drugs [62]. Hamilton [63] reported that by binding with FK506-binding protein (FKBP), immunosuppressive agents, particularly tacrolimus and its analogues, could produce neuroprotective and neurogenerative effects. These small molecular immunosuppressive drugs are thus able to cross the BBB easily and are useful in treating brain and spinal cord injuries [53]. Peptidomimetics are small protein-like chains intended to mimic peptides [64]. There are chemical modification methods that involve modifying the peptide structure to improve pharmacokinetic properties while simultaneously retaining a specific amino acid part(s) responsible for activity [64]. The absorption of polar drugs can be achieved by increasing their hydrophobicity, however, the volume of distribution of the drug within the body will also increase [53,65]. The transcellular pathway (Figure 2A) allows the passive diffusion of small, lipophilic molecules through the BBB and into the brain, while carrier-mediated transport (Figure 2B) is a type of facilitated transport that employs specific proteins to move molecules from the environment into and through the cell [66]. Endocytosis and transcytosis (Figure 2C,D) are receptor-mediated drug delivery processes that aid in the uptake of molecules including drugs across the BBB [53]. Endocytosis and transcytosis are regulated by receptors including the insulin receptors, transferrin receptor, low-density lipoprotein receptor-related protein (LRP), neonatal Fc receptor present in the brain and the transferrin receptor [53]. Angiopep-2 is a synthetic peptide and a ligand for LRP1 receptors which is readily transported across the BBB. Conjugating drugs such as doxorubicin onto PEGylated oxidized multi-walled carbon nanotubes (O-MWNTs) modified with angiopep-2 (O-MWNTs-PEG-ANG) has been shown to enhance the uptake of doxorubicin into the brain [67]. Endocytosis of materials can occur either through phagocytosis or pinocytosis [53].

**Table 1:** Non-invasive approaches for the delivery of drugs across the BBB.

Approach	Drug Delivered across the BBB	Observations	Ref.
Intranasal drug delivery	α-L-idur-onidase (IDUA) encoding adeno-associated virus serotype 9 (AAV9) vector	Intranasal administration of α-L-idur-onidase (IDUA) encoding adeno-associated virus serotype 9 (AAV9) vector results in enzyme diffusion into deeper areas of the brain and reduction of tissue glycosaminoglycans storage materials in the brain.	[68]
Altered drug solubility	Doxorubicin	Conjugation of doxorubicin with angiopep-2 increased delivery of doxorubicin to the brain and showed good bioavailability and lowtoxicity.	[67]
NP drug delivery system	Zidovudine	Zidovudine was delivered via nanostructured lipid carriers into an in vitro human brain cell line (C6) and led to a significantly higher accumulation of the drug in the brain cells. The results suggest that these NPs could be a promising delivery system to enhance the brain uptake of zidovudine and other non-nucleotide ARVs.	[69]
NP drug delivery system	Atazanavir	In vitro delivery of atazanavir by solid lipid NPs into a hCMEC/D_3_ cell line demonstrated a significantly higher drug accumulation compared to the drug aqueous solution alone.	[70]
Polymer drug conjugates	Ciprofloxacin	Increase in the uptake of PEGylated ciprofloxacin when the surfaces of the biologically active polymer core/shell NPs were modified with Tat peptide (TAT–PEG-*b*-Chol nanoparticles).	[71]
Peptidomimetics	HAYED peptide	A 16 lysine (K16) residue-linked low-density lipoprotein receptor-related protein (LDLR)-binding amino acid segment of apolipoprotein E (K16APoE) was used to deliver a therapeutic peptide (HAYED) into an Alzheimer’s disease mouse model brain leading to reduced necrosis.	[72]
Viral vectors	Gadoteridol	Gadoteridol was co-infused with adeno-associated viral type 2 vectors and results showed that infusion of therapies directly into the disease- infected regions of the human brain with convection-enhanced delivery provides an effective strategy for treating neurological disorders.	[73]
Trojan horse approach	HIRMAb-IDUA fusion protein	HIRMAb-IDUA fusion protein, also called valanafusp alpha has been administered to patients with mucopolysaccharidosis (MPS) I. Patients were treated with HIRMAb-IDUA weekly by IV infusion for over a year. MPS I patients treated with HIRMAb-IDUA who suffered from severe mental retardation demonstrated stability in their IQ from further decline.	[74]

The permeability of the BBB can be increased through use of pharmacological agents thus enabling cells to become more permeable. In view of the fact that the BBB controls material, nutrients and cell transfer from the blood to the brain and from the brain to the blood [7,75], vascular permeability is related to BBB permeability. Histamine and vasoactive peptides are agents responsible for inflammatory reactions causing a temporal increase in vascular permeability and vascular leakage [76]. The vasodilator, bradykinin, increases vascular permeability by acting on bradykinin 2 receptors [53]. A 9-amino-acid peptide, labradimil (Cereport^®^; also known as RMP-7), is a formulated drug delivery system that shows selectivity for the bradykinin β_2_ receptor designed to increase the permeability of the BBB. In-vitro studies have revealed that labradimil selectively binds to bradykinin β_2_ receptor, has a longer plasma half-life than bradykinin, and initiates bradykinin-like second messenger systems such as an increase in the turnover of intracellular calcium and phosphatidylinositol [77]. Observations using electron microscopy showed that intravenous labradimil increases the permeability of the BBB by loosening the tight junctions of the endothelial cells [77]. The success of disrupting the BBB depends on the space created in the pores being large enough to permit the entry of molecules without damaging the structure of the cell [53].

BBB pores are typically <1 nm; however, particles that are several nanometers in diameter can also cross the BBB via carrier-mediated transport [78]. Any non-specific pores in the paracellular space nullifies the ability of the endothelial barrier to effectively regulate molecules and ions across the barrier. In essence passive permeability across the paracellular space would result in a short-circuit in the regulation of the BBB [79]. Tight junctions have a size-selective permeability to uncharged particles of up to 4 nm and low permeability to larger particles [7]. This means that each tight junction forms a 4 nm pore and that molecules larger than 4 nm would pass through gaps in the junctions. NPs however may utilize a specific alternative lipophilic mechanism to cross the BBB [79]. NPs larger than 4 nm can cross the BBB. Adams et al. [80] successfully delivered fibrin γ377–395 peptides conjugated to iron oxide (Fe_2_O_3_) NPs of 21 ± 3.5 nm in diameter to inhibit microglial cells in rTg4510 tau-mutant mice in-vivo [80,81]. Otani and Furuse. [82] reported that the size-selective pathway of tight junctions is approximately 60 Å (equivalent to ~6 nm). The permeability of the BBB is more dependent on the molecular properties of the molecule than its size [7]. Small molecules can cross the BBB via lipid-mediated free diffusion, provided that they have a molecular weight of <400 Da and form less than 8 hydrogen bonds [60,83,84,85]. Thus, lipophilic molecules can cross the membranes of the BBB even if their size is large. Typically, molecules less than 250 nm in diameter are taken up effectively by cells in the brain [86,87]. However, the size of the NP that can cross the BBB greatly depends on the location of the brain (i.e., the pathway for crossing the BBB) and target tissue at the brain site [87].

Nanoporation is a type of electroporation that generates very small holes (<2 nm) in plasma membranes [88]. The pores formed are transient enabling transcellular drug uptake as opposed to an opening of tight junctions which is typical with non-invasive methods. Thus, there is no alteration of brain endothelial cells and the formation of gaps in the endothelial junction. Sridhara and Joshi [89] studied the poration dynamics of lipid translocation driven by nanoporation due to multiple high-intensity (>100 kV/cm), ultrashort electrical pulses and to determine whether the pores, if formed, could remain open even after the electrical field had ceased. In their molecular dynamics (MD) simulations, the water–membrane system contained 37,157 water molecules and 512 dipalmitoylphosphatidylcholine (DPPC) lipid molecules for a total of 137,071 atoms in a 12.948 nm × 12.999 nm × 10.364 nm simulation box. The MD results displayed a gradual pore creation that began during the ‘ON-time’—start of the first pulse of the unruptured membrane patch. The existence of the first small nanopore was observed at the time (*t*) = 5 ns which grew larger by the 10 ns time instant. During the termination of the electrical pulse at 10 ns and 60 ns, the pore remained open without considerable changes or reduction. The pore was seen to be at its largest at the end of the second pulse. The results of this study coincide with the experimental study results of Pakhomov and colleagues [90] using 600 ns pulses that have shown that nanopores are stable for many minutes. The use of multiple pulsing along with higher applied voltages could result in a larger pore density [89]. A larger pore density would be beneficial to pore coalescence and may promote the appearance of larger sized entry sites at the plasma membrane [89]. Sridhara and Joshi [89] concluded that once nanopores are formed, they can remain open for long periods of time (there is no record of the duration of time the pores remained open in the study—the pore was still open at the beginning of the second electric pulse and there is no record of pore closure after *t* = 70 ns). The pore effects are expected to be much stronger with multiple pulsing. Nanopores that are stable for many minutes could significantly have an impact on cell electrolyte and water balance [89,91]. Multiple nanosecond duration electric pulses (nsEPs) cause rapid cell swelling and blebbing (bulging out of the cell membranes), while substances such as digitonin (a mild detergent that permeabilizes plasma membranes) eradicates swelling and causes blebs to collapse [90]. To date, most of the research has focused on controlled measurements using artificial lipid bilayer structures or indirect methods of nanoporation detection. Although both techniques provide useful insights, they are unable to directly detect and describe the dynamic nature of the poration and the recovery process in the affected living cells [91,92].

### 2.3. Application of MENs to Deliver Drugs across the BBB for CNS TB and HIV

NPs are particles in the nanoscale range between 1 to 100 nm (in at least one dimension) whose properties vary depending on their size, surface area, uniformity, optical properties and functionalization [93]. NPs are effective delivery systems for a variety of payloads [60,94]. NPs are being investigated towards the delivery of drugs for infectious diseases [95]. Apart from demonstrating good stability and tunability to carry cargo, NPs can cross biological barriers, providing controlled and sustained therapeutic effects at target sites [96].

MENs are a class of NPs that exhibit magnetic and electric properties that can be controlled utilizing magnetic and electric fields. MENs are typically 30 nm in diameter, however, these NPs can be as large as 600 nm [97]. Calcination approaches have been investigated to determine the sizes of MENs. Hadjikhani et al. [97] investigated the effect of alteration of calcination temperatures on the size of MENs. The authors reported that calcination at 600 °C resulted in 30 nm MENs, 700 °C for 100 nm MENs, 780°C for 200 nm MENs and 850 °C for 600 nm MENs. Thus, the higher the calcination temperature, the larger the size of the MENs [97]. MENs are comprised of a lattice crystal structure with a magnetostrictive cobalt ferrite (CoFe_2_O_4_) core surrounded by a barium titanate (BaTiO_3_) piezoelectric shell. The tetragonal crystal structure of the BaTiO_3_ shell and the cubic structure of the CoFe_2_O_4_ core has been shown using X-ray diffraction (XRD) [30]. Microscopy images of MENs show an irregular-sphere-like morphology [98,99]. MENs offer advantages in that they are able/show potential to (i) achieve targeting driven by an external magnetic force, (ii) provide on-demand externally controlled drug release and (iii) provide image-guided precision drug delivery [30]. These properties are advantageous towards drug delivery in the CNS for TB and HIV.

**Figure 3:**
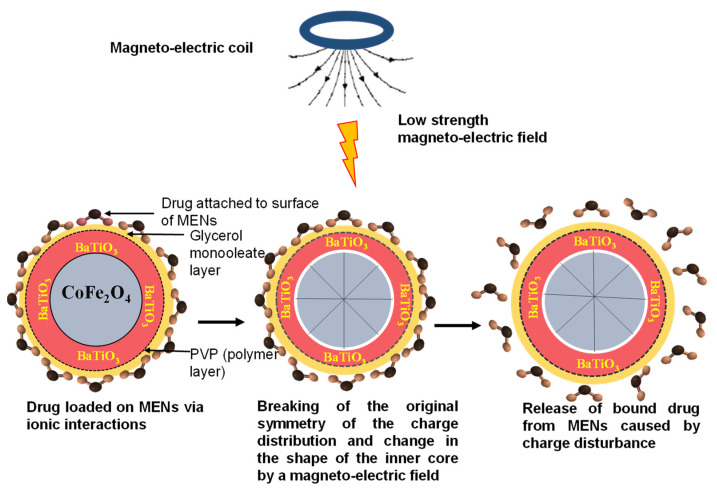
Schematic diagram of the effect of an applied magneto-electric field on core-shell MENs. MENs surrounded by a polyvinylpyrolidone (PVP) polymer layer and glycerol monooleate layer with a drug loaded on the surface of MENs. Upon the introduction of low strength magneto-electric field, the original symmetry of the charge distribution breaks and the shape of the inner core of MENs changes (i.e., magnetostriction) and changes the shape of the piezoelectric shell resulting in the release “shaking off” of the drug bound to MENs caused by charge disturbance.

Externally applied magnetic fields can change the shape of the inner core of MENs. This phenomenon is known as magnetostriction and changes the shape of the piezoelectric shell (Figure 2E). The change in the shape of the piezoelectric shell creates an electric field at the surface of the MEN which induces temporal nanoelectroporation of the cells whereby holes less than 2 nm in the cell plasma membranes contributing to enhanced drug permeation and uptake of MENs in the diseased cells [61,100]. From the literature, it is unclear whether phagocytosis/endocytosis is also a major uptake route for MENs, or whether MENs also transiently alter the size of the paracellular tight junctions across the BBB. The greater the magnetic field, the greater the electric field that is produced [101,102]. Typically, diseased cells have a different electroporation than normal cells and, have a lower threshold for electroporation and are therefore more permeable when exposed to an electric field [101,102]. Electroporation has been detected using dyes (fluorophores or color stains) or functional molecules by measuring the efflux of biomolecules, monitoring cell swelling and through conductivity measurements, impendence measurements and voltage-clamp techniques [103]. Any other substance present at the blood-side of the BBB would also be taken up across the cells due to the altered permeability. Drug release has been described to be achieved through “shaking off” of the drug on the MENs as the induced alternating magnetic field shifts the magnetic dipole of the particle the drug is released intracellularly (Figure 3) [101].

MENs have been investigated for drug delivery for CNS HIV. Nair et al. [104] demonstrated the in-vitro on-demand release of zidovudine (AZTTP) from 30 nm CoFe_2_O_4_ @ BaTiO_3_ MENs by applying a low alternating current magnetic field. Nair et al. [104] demonstrated that the MEN-AZTTP nanoformulation showed 100% drug release at low alternating current (44 Oe at 100 Hz) without losing drug integrity and further showed HIV-p24 inhibition in vitro with good transendothelial BBB transmigration efficiency. An in vitro BBB model made from layers of brain microvascular endothelial cells (BMVEC) on the one side and astrocytes on the other side was used in this study. Approximately 40% of the MENs-AZTTP could penetrate the BBB under low-energy direct current magnetic field, this is three times higher than the free drug [104]. An alternating current (AC)-magnetic field generated through electromagnetic coils as external stimuli were used to demonstrate the on-demand release of AZTTP (100% at 64 Oe). The AC-magnetic field could cause polarization changes on the MENs surface bringing about the release of AZTTP without hindering efficacy. The authors also showed that AC-magnetic field stimulation on MENs produced a localized electric field and sufficient ultrasound to open the cell membrane pore and deliver the MENs-AZTTP complex intracellularly. According to an investigation performed by Kaushik et al. [105] MENs (50µg) and AC-magnetic field (60 Oe) do not affect the viability of brain microglial cells. In this study, the uptake of MENs into the cells was demonstrated through nanoelectroporation using ac magnetic field stimulation. MENs were taken up by microglial cells without affecting the health of the cell (viability > 92%) following optimization of AC- magnetic field at 60 Oe at 1 kHz [105]. Furthermore, focused ion beam transmission electron microscope (FIB-TEM) analysis of microglial cells demonstrated a non-agglomerated distribution of MENs inside the cell and no loss of their elemental crystalline characteristics [105]. Pandey et al. [98] demonstrated that MENs displayed enhanced cell uptake (via microglial brain cells) and controlled drug release. The authors further confirmed that the AC-magnetic field stimulated at 60 Oe confirmed the localized surface potential enhancement of MENs.

In another study, the delivery of Beclin1siRNA across the BBB using MENs was demonstrated by Rodriguez et al. [106] towards control of the inflammatory effect of HIV infection. CoFe_2_O_4_ @ BaTiO_3_ NPs bound to small interfering ribonucleic acid (siRNA) targeting Beclin1 to cross the BBB and to decrease the neurotoxic effects of HIV-1 infection in the CNS brought by an on-demand release of siRNA using an in vitro primary human BBB model [106]. The result of this research showed that Beclin1siRNA attached to MENs was released through AC-magnetic stimulation [106].

MENs can be designed with a coating layer. As can be seen in chromites, ferromagnetic materials due to their magnetic nature, have a high tendency to agglomerate [107,108]. To avoid agglomeration, the NPs can be coated or dispersed in a non-magnetic matrix [107]. Coating layers consisting of either glycerol monooleate (GMO), polyethylene glycol, or poly-L-Lysine have been reported [101]. Kaushik et al. [109] reported the effective controlled on-demand release of a nanoformulation composed of clustered regulatory interspaced short palindromic repeat (CRISPR)—associated 9, also known as Cas9/gRNA bound with MENs across the BBB to inhibit latent HIV-1 infection in microglial (hµglia)/HIV (HC69) cells. This approach led to the eradication of latent HIV in the cells [109]. While the above studies demonstrate efficacy in vitro, there are no studies demonstrating the effectiveness of this approach using in vivo animal models.

To date, no studies have been reported on the application of MENs for delivery of drugs for eradication of *M. tb* in a CNS infection model. However, from reports on the delivery and efficacy of HIV drugs such as zidovudine delivered using MENs, we can expect similar observations for TB drugs.

## 3. Conclusions and Future Directions

In this manuscript we have discussed the challenges faced by drugs for CNS TB and HIV in crossing the BBB, approaches to enhance drug delivery into the CNS and the application of MENs for this purpose. We have found that MENs show promise as effective systems for non-invasive drug delivery and therapy across the BBB. These NPs provide an on-demand externally controlled drug release and have demonstrated efficacy in CNS HIV model studies. However, significant further work is required towards the translation of this technology to the clinic. Apart from a need for proof-of-concept studies in animal models of HIV and TB, additional insights into the interaction of the MENs with the biological system are required. Understanding of achievable controlled drug release and intracellular and in vivo pharmacokinetic profiles of the drug, would be crucial for diseases such as HIV and TB where sustained drug concentrations above the minimum effective concentration are required. Indeed, significant efforts are directed at long-acting nano ART formulations, and some have reached the clinic [110]. Toxicity studies over extended periods of time, investigations of the short-and long-term effects of AC-fields and nanoporation on the brain are also required. The intracellular trafficking and biodegradation of the MENs should be studied further and long-term toxicity data generated.

## Data Availability

Not applicable.
